# Magnitude and associated factors of adverse perinatal outcomes among women with oligohydramnios at 3rd trimester at University of Gondar comprehensive specialized hospital, North West Ethiopia

**DOI:** 10.3389/fgwh.2022.958617

**Published:** 2023-01-30

**Authors:** Mezigebu Molla, Zelalem Mengistu, Winta Tsehaye, Getasew Sisay

**Affiliations:** Department of Gynecology and Obstetrics, School of Medicine, College of Medicine and Health Sciences, University of Gondar, Gondar, Ethiopia

**Keywords:** oligohydramnios, adverse perinatal outcome, gondar, Ethiopia, oligohydraminos, adverse perinaqtal outcome

## Abstract

**Background:**

Oligohydramnios is a pregnancy condition characterized by low volume of amniotic fluid. Based on ultrasound measurement, it is defined as a single maximum vertical pocket of liquor less than 2 cm or summation of four quadrants vertical pockets of liquor measurement less than 5 cm. It is associated with multiple adverse perinatal outcomes (APO) and complicates 0.5%–5% of pregnancies.

**Objective:**

To assess magnitude and associated factors of adverse perinatal outcome among women with oligohydramnios at 3rd trimester at University of Gondar Comprehensive Specialized Hospital, North West Ethiopia

**Methods:**

Institution based cross-sectional study was employed from April 1 to September 30, 2021 in which 264 participants were involved. All women with oligohydramnios at 3rd trimester who meet the inclusion criteria were included. Semi- structured questionnaire was used for data collection after Pre-tested. Collected data was checked for completeness; clarity then coded and entered using Epi data version 4.6.0.2 then exported to STATA version 14.1 for analysis.

**Result:**

The magnitude of APO was 46.6% (95%CI: 40.5–52.7%). Null parity [AOR =  2.2, 95%CI (1.2–4.2)], presence of hypertensive disorders of pregnancy (HDP) [AOR = 4.9, 95%CI (2.0–12.1)] and presence of intrauterine growth restriction (IUGR) [AOR = 8.4, 95%CI (3.5–20.2)] were found to be predictors of APO.

**Conclusion:**

Third trimester oligohydramnios is associated with APO. The presence of HDP, IUGR and being nulliparous were predictors of APO.

## Introduction

Amniotic fluid (AF) is necessary for normal development of fetal respiratory, gastrointestinal, urinary tract, and musculoskeletal systems. It is also important for the continued fetal growth in a nonrestricted, sterile and thermally controlled environment (1, 2). AF volume is affected by varies conditions such as postmaturity syndrome; maternal diseases like hypertension, diabetes in pregnancy (especially poorly controlled), autoimmune disorders; maternal medications (prostaglandin synthetase inhibitors); altitude; fetal anomalies (including both immune and nonimmune fetal hydrops); and fetal weight (macrosomic and growth restricted) (1–3).

At third trimester the volume of AF is from fetal urination, lung secretion, fetal swallowing, and intramembranous absorption (4).

Oligohydraminos is usually diagnosed based on ultrasound measurement of amniotic fluid volume and considered when amniotic Fluid Index (AFI) is less than 5 cm or a single deepest pocket of amnionic fluid is below 2 cm (2, 3). Oligohydramnios at third trimester affects 1%–5% of pregnancies (5, 6).

Complications associated with oligohydramnios depend on the gestation at which the diagnosis is made. If the diagnosis of oligohydraminos early in pregnancy, it is often associated with more serious complications such as compression of fetal organs resulting in birth defects, pulmonary hypoplasia and increased risk of miscarriage or stillbirth. Whereas latter in pregnancy, the complications are IUGR, preterm birth, intra uterine fetal demise, intra-partum fetal distress and birth asphyxia. During labor, it can cause cord compression, meconium-stained fluid, abnormal fetal heart rate, operative interventions, and increased risk of cesarean delivery, lower Apgar scores, intensive care unit admission and neonatal death (2, 3, 7–11).

Oligohydraminos associated composite adverse perinatal outcomes varying between 9.7%–38.1% (6, 12). Meconium stained amniotic fluid (MSAF), non-reassuring fetal heart rate pattern (NRFHRP), prematurity, low birth weight, low 5th minute Apgar score, birth asphyxia, IUGR, respiratory distress, early neonatal death and still births are some of the adverse perinatal outcomes associated with oligohydraminos (9, 13–18).

Occurrence of adverse perinatal outcomes in oligohydraminos depends on the extent of oligohydramnios, and the presence of other factors like post term pregnancy, IUGR, presence of hypertensive disorders of pregnancy, gravidity, ANC booking, non-reactive NST, Gestational age at delivery and the cause of oligohydramnios (6, 19–25).

There has been a trend of delivering a fetus if diagnosis of oligohydraminos made at third trimester of pregnancy fearing occurrence of adverse perinatal outcomes. There is also dilemma about the route of delivery especially in set ups where there is no intrapartum electronic fetal heart rate monitoring like Ethiopia where this study was done (12–13, 26–30). There is scarcity of evidences which assess the perinatal outcomes associated with oligohydramnios at third trimester.Thus, this study was aimed to assess the magnitude and associated factors of adverse perinatal outcomes among women with oligohydramnios at third.

## Materials and methods

### Study design, period and setting

Institution based cross-sectional study was employed among pregnant mothers with oligohydramnios at third trimester who were attended at university of Gondar comprehensive specialized hospital from April to September 2021.University of Gondar is one of the pioneer teaching hospitals in North West Ethiopia in previous capital of Ethiopia, Gondar city. The hospital gives service for more than 7 million people in its catchment and has 700 beds of which 177 beds belonged to obstetrics and gynecology services. Its ANC clinic and labor ward provide care for 9,000 and 900 pregnant women monthly respectively.

### Study population and inclusion/exclusion criteria

All pregnant women who had oligohydramnios at 3rd trimester and attended maternity unit of university of Gondar comprehensive specialized hospital were included. Whereas women with PROM [*N* = 15], who cannot communicate, mentally and severely ill, diagnosed IUFD at presentation, with lethal congenital anomaly [*N* = 3], with unknown or unreliable date or with absence of early milestones to date the pregnancy [*N* = 22] were excluded.

### Sample size determination

The sample size was estimated using single population proportion formula. By considering, 95% confidence level, 5% margin of error, composite adverse perinatal outcome of 38.1% in similar setting (6) and 10% of non-responsive rate the final sample size became 398.

### Sampling technique and procedure

A consecutive sampling technique was used to include all women with oligohydramnios at 3rd trimester who meet the inclusion criteria during the study period.

### Study variables

After the identification of patients fulfilling the inclusion criteria, baseline data were collected from the patients and clinical variables which cannot be provided by the patient were retrieved from medical records.

Oligohydramnios was considered when AFI ≤ 5 cm or a single deepest pocket of amniotic fluid ≤ 2 cm.

Adverse perinatal outcome was considered if one of the following present Low Apgar score (5th Apgar score < 7), low birth weight, neonatal intensive unit (NICU) admission, perinatal death, MSAF, NRFHR, perinatal asphyxia.

### Data collection procedures

Data collection was made by trained medical interns and obstetrics and gynecology residents using Amharic pretested semi-structured questionnaires adapted from similar researches. Written informed consent of participants were obtained after the purpose of the study was clearly explained then Interviewer administered questionnaire.

### Data quality assurance

The questionnaire was first developed in English and translated into Amharic language then back to English and consistency was checked. Two-days training was made to the data collectors and 5% questionnaires were pretested on similar setting. The training session covers the overall objective of the study, definition of terms and concepts, approaching of respondents, data collection tools and techniques of interviewing. The collected data was also cross-checked for its completeness, consistency, accuracy, and clarity on a daily basis. Daily supervision of data collectors was carried out throughout data collection period.

### Data processing and analaysis

Data was coded and entered using epi data version 4.6.0.2 then exported to STATA version 14.1. Further data clean-up for completeness clarity and recoding was done on STATA before the actual analysis. Descriptive statistics like frequency, percentage, mean, and standard deviation was done to describe the study population in relation to different variables. A chi-square test was done for all variables to check for the assumptions. Stepwise Logistic regression analysis was carried out to identify the independent factors associated with adverse perinatal outcome. Variables having *p*-value ≤ 0.2 from the bivariable analysis was included for the final multivariable logistic regression and variables having *P*- value <0.05 was considered to have a significant association with adverse perinatal outcome. An adjusted odds ratio with 95% CI was used as a measure of association.

### Ethical considerations

The ethical clearance was obtained from the ethical review committee of the school of medicine, College of medicine and health sciences, on behalf of Institutional Review Board (IRB) of university of Gondar(Dr. Mulugeta Ayalew, Director of school of Medicine, reference number: 643/2021).

## Results

### Socio-demographic characteristics of the study participants

Two hundred sixty four women participated in the study. Three-fourth of the participants were from the urban setting [*N* = 198, 75%] and majority were within the age group of 20–34 years [*N* = 225, 85.29%]. The mean age was 27.36 ± 4.85 years [18–40 years] (Table 1).

**Table 1:** Sociodemographic characteristics of participants, 2021 [*N* = 264].

Variable	Participant	Frequency	Percentage
Age	18–19	5	1.89
	20–34	225	85.23
	35–40	34	12.88
Residence	Rural	66	25.00
	Urban	198	75.00
Religion	Orthodox	244	92.42
	Muslim	16	6.06
	Others	4	1.52
Marital status	Married	256	96.97
	Unmarried*	8	3.03
Educational level	Unable to read and write	30	11.36
	Primary school	39	14.77
	Secondary school	76	28.79
	College and above	119	45.08
Occupation	Housewife	115	43.56
	Farmer	21	7.95
	Student	16	6.06
	Merchant	49	18.56
	Non/gov employee	63	23.86
Monthly family income in birr	Income ≤5000	88	33.33
	Income 5,000–10,000	108	40.91
	Income 10,000–15,000	52	19.70
	Income >15,000	16	6.06

* Unmarried – those who have no legalized husband during the study period (widowed, divorced, single, separated).

### Obstetric characteristics of participants

Nearly half of the study participants were nulliparous (51.5%). At delivery forty percent of participants (39.8%) were full term; 14.4% were post term and 6.8% were preterm. The mean gestational age at diagnosis of oligohydraminos was 40 ± 2 weeks [30 + 4–44 weeks] (Table 2).

**Table 2:** Obstetric characteristics of study participants, 2021 [*N* = 264].

Variable	Participant	Frequency	Percentage
Parity	Nulliparous	136	51.52
	Multiparous	128	48.48
	Total	264	100.00
Gestational age	28–36 + 6 weeks (Preterm)	18	6.82
	37–38 + 6 weeks (Early term)	56	21.21
	39–40 + 6 weeks (Full term)	105	39.77
	41–41 + 6 weeks (Late term)	47	17.80
	≥42 (Post term)	38	14.39
	Total	264	100.00
ANC	Yes	262	99.24
	No	2	0.76
	Total	264	100.00
Place of ANC follow-up	Local health center	132	50.38
	Private clinic	53	20.23
	Primary hospital	9	3.44
	University of Gondar hospital	68	25.95
	Total	262	100.00
Number of visits	<5	96	36.64
	≥5	166	63.36

### Antepartum obstetric and medical problems

Among participants 62.1% were having antepartum obstetric problems like IUGR (22.4%), HDP (17.1%), post-term (14.4%), and previous cesarean scar (12.9%). Fourteen (5.3%) participants had medical problems such as malaria (five cases), anemia (three cases), HIV/AIDS (three cases), hyperthyroidism (two) and one case with epilepsy. Majority (78.8%) of the participants had amniotic fluid index of 2 cm–5 cm.

At admission three-fourth of the participants had reactive non stress test (NST), and 25% of them had non-reactive NST. Umbilical artery Doppler study was done for 112 participants and 77.7% were normal and 22.3% were abnormal study. Among the abnormal Doppler findings six (24%) were absent end diastolic flow while 76% were abnormal Doppler (Table 3).

**Table 3:** Antepartum obstetric and medical problems among participants, 2021.

Variables	Participants	Frequency	Percent
Hypertensive disorders of pregnancy	Yes	45	17.05
Antepartum hemorrhage	Yes	7	2.65
Post term	Yes	38	14.39
Malpresentation	Yes	24	9.09
Previous cesarean scar	Yes	34	12.88
Intrauterine growth restriction	Yes	59	22.35
	No	100	37.88
	Total	264	100.00
Amniotic fluid index	<2.0	56	21.21
	≥2.0–5.0	208	78.79
	Total	264	100.00
Admission CTG	Reactive	198	75
	Non-reactive	66	25
	Total	264	100.00
Doppler us	Yes	112	42.42
	No	152	57.58
	Total	264	100.00
Doppler finding	Normal	87	77.68
	Abnormal	25	22.32
	Total	112	100.00
Abnormal doppler	Umbilical artery Absent end diastolic flow	6	24
	Pathologic cerebroplacental ratio	19	76
Medical problems	Yes	14	5.30
	No	250	94.70
	Total	264	100.00

### Mode of delivery

Labor started spontaneous in 28.0%, and induced in 47.4% with trans-cervical balloon catheter and oxytocin. Cesarean section (CS) was done for 59.1% of the participants of which 40% had elective CS.There was no cesarean delivery for the mere presence of oligohydraninos alone (Tables 4, 5).

**Table 4:** Type of labor and mode of delivery of participants, 2021 [*N* = 264].

Type of labor	Mode of delivery			
	Cesarean delivery	Vaginal Delivery	Operative vaginal delivery	Total
Spontaneous	33 (21.15%)	38 (36.54%)	3 (75%)	74 (28.03%)
Induced	58 (37.18%)	66 (63.46%)	1 (25%)	125 (47.35%)
No labor (Elective CD)	65 (41.67%)	–		65 (24.62%)

**Table 5:** Indications for cesarean delivery among participants, 2021

Indication	Frequency	Percent
Previous CS scar	25	16.03
Malpresentation	21	13.46
NRFHRP	63	40.38
MSAF	12	7.69
failed induction	10	6.41
IUGR with affected doppler, NR_NST &other factors precluding induction	17	10.90
poor progress in labor precluding augmentation	3	1.92
CPD	2	1.28
Oligohydramnios + Elderly primigravida	3	1.92
Total	156	100.00

### Perinatal outcome

The rate of adverse perinatal outcome was **46.6% [95%CI, 40.5–52.7%**]. The frequency of adverse outcomes were low birth weight (19.3%), non-reassuring fetal heart rate pattern (25.4%), neonatal admission (26.1%), Meconium-stained amniotic fluid (23.1%), low 1st minute APGAR (2.3%), perinatal asphyxia (1.5%) and 4 perinatal deaths. Among the perinatal deaths, one was intrapartam death; the three were early neonatal deaths. Sixty-nine neonates were admitted to NICU with diagnosis of RDS [*N* = 25, 36.23%], MAS [*N* = 18, 26.08%], neonatal sepsis [*N* = 23, 33.33%] (Table 6).

**Table 6:** Adverse perinatal outcomes of participants, 2021.

Variables	Yes/No	Frequency	Percent
Birth weight in kg	<2.5 kg	51	19.32
	≥2.5 kg	213	80.68
Non reassuring fetal heart rate pattern	Yes	67	25.38
	No	197	74.62
Neonatal admission	Yes	69	26.14
	No	195	73.86
Meconium-stained amniotic fluid	Yes	61	23.11
	No	203	76.89
1st min APGAR	<7	6	2.27
	**≥7**	258	97.73
Perinatal death	Yes	4	1.52
	No	260	98.48
PNA	Yes	4	1.52
	No	260	98.48

### Factors associated with adverse perinatal outcome

Adverse perinatal outcomes were increased by two-fold in nulliparous compared to multiparous [AOR = 2.2, 95%CI: 1.2–4.2]. Mothers whose pregnancy complicated with IUGR had eight-fold increase risk of adverse perinatal outcomes compared to mothers with no IUGR [AOR = 8.4, 95%CI: 3.5–20.1]. Similarly in women with hypertensive disorders of pregnancy the odds of experiencing adverse perinatal outcome were found to be increased by nearly five-fold as compared to women with no hypertensive disorders of pregnancy [AOR = 4.933, 95%CI: 2.015–12.077] (Table 6, 7).

**Table 7:** Bivariate and multivariable analysis of factors associated with adverse perinatal outcome, 2021.

Variables	Adverse Perinatal outcome				
	No	Yes	COR [95%CI]	AOR [95%CI]	*P* value
Age in years					
18–19	3 (2.13%)	2 (1.63%)	0.697 (0.114–4.25)	0.543 (0.053–5.547)	
20–34	115 (81.56%)	110 (89.43%)	1	1	
35–40	23 (16.31%)	11 (8.94%)	0.5 (0.23–1.074)	0.617 (0.233–1.636)	
Income in ETB Per month					
≤5,000	41 (29.08%)	47 (38.21%)	3.349 (1.029–11.494)	3.662 (0.89–15.076)	0.072
5000–10,000	60 (42.55%)	48 (39.02%)	2.4 (0.728–7.91)	2.985 (0.754–11.811)	0.119
10,000–15,000	28 (19.86%)	24 (19.51%)	2.571 (0.732–9.03)	2.475 (0.586–10.451)	0.218
>15,000	12 (8.51%)	4 (3.25%)	1	1	
GA in weeks					
28–36 + 6 (Preterm)	2 (1.42%)	16 (13.01%)	15.333 (3.34–70.403)	3.801 (.597–24.22)	.158
37–38 + 6 (early term)	25 (17.73%)	31 (25.20%)	2.377 (1.224–4.614)	1.422 (.627–3.224)	.399
39–40 + 6 (full term)	69 (48.94%)	36 (29.27%%)	1	1	.
41–41 + 6 (late term)	25 (17.73%)	22 (17.89%)	1.687 (.837–3.398)	1.759 (.784–3.949)	.171
≥42 (post term)	20 (14.18%)	18 (14.63%)	1.725 (.812–3.665)	2.244 (.928–5.426)	.073*
Marital status					
Married	151 (98.69%)	105 (94.59%)	1	1	
Unmarried	2 (1.31%–)	6 (5.41%)	4.314 [0.854–21.791]	1.066 [0.159–7.157]	0.948
Parity					
Nulliparous	60 (42.55%)	76 (61.79%)	2.183 [1.332–3.576]	2.24 [1.209–4.15]	0.01**
Multiparous	81 (57.45%)	47 (38.21%)	1	1	.
HDP					
Yes	10 (7.09%)	35 (28.46%)	5.21 [2.45–11.06]	4.933 [2.015–12.077]	.001***
No	131 (92.91%)	88 (71.54%)	1	1	.
IUGR					
Yes	9 (6.38%)	50 (40.65%)	10.05 [4.67–21.59]	8.395 [3.496–20.16]	0.001***
No	132 (93.62%)	73 (59.35%)	1	1	
AFI					
<2.0 cm	19 (13.48.03%)	37 (30.08%)	2.763 (1.489–5.126)	1.839 (0.863–3.921)	0.115
≥2.0 cm–5.0 cm	122 (86.52%)	86 (69.92%)	1	1	.
NST					
Reactive	119 (84.40%)	89 (72.36%)	1	1	.
Non–reactive	22 (15.60%)	34 (27.64%)	2.066 (1.131–3.775)	1.694 (.798–3.597)	0.17

* *p* < .1.

** *p* < .05.

*** *p* < .01.

## Discussion

In this study 264 study participants were involved and majority were in the age group of 20–34 years [*N* = 225, 85.3%] with mean age being 27.4 ± 4.9 years [18–40 years] which is similar with study done in Mekele, Ethiopia(85.3% vs. 89.4%) (6). This shows that most of the oligohydraminos were among women whose age group is said to be favorable for better pregnancy outcomes. The gestational age was full term for 40% of participants, and the mean gestational age was 40 ± 2 weeks [30 + 4 – 44 weeks] which showed most of the deliveries were in gestational age group assumed to have at lower risk to develop adverse perinatal outcomes. Slightly more than half (51.0%) of the participants were nulliparous, this might show most of the participants were at increased risk for developing nulliparous related obstetric problems and labor abnormalities.

Labor was spontaneous in 28.0% and induced in 47.4% of the participants. Of all participants 59.1% delivered by cesarean and 39.4% were vaginal. This shows that the presence of oligohydramnios is a major risk for having cesarean delivery. However, it is lower than previous studies with CD rate varied 69.9–80.3%, and vaginal delivery varied [19.7–30.1% (5, 6)].

In this study the rate of adverse perinatal outcome was **46.6%**. Compared to similar study done in Israel the rate of composite adverse outcome was found to be higher [9.7%]. (12). This can be explained by the difference in obstetric profile of study participants as the study in Israel included only cases with isolated oligohydramnios at term. This difference might also be explained by the study in Israel uses fetal PH assessment as an adverse outcome and this may reduce cases of NRFHR pattern while in our study we have no PH assessment and may increase cases of NRFHRP.

This finding was also higher than similar study done in Ethiopia [38.1%] (6). This can be explained by the high number of cases with other obstetric problems HDP [17.05 vs. 6.2%], IUGR [22.35 vs. 11.72%] in this study. This may also be explained by the high number of NRFHR cases in our study which can be detected with CTG monitoring.

The rate of individual adverse outcomes also found to be higher than previous study done in Ethiopia, NICU admission rate [26.14 vs. 15.4%], NRFHRP [25.38 vs. 15.8%], MSAF [23.11 vs. 9.2%] while LBW almost similar [19.32 vs. 19.8%]. This can be explained by better detection of fetal heart rate abnormalities with CTG and better antepartum fetal evaluation with maternal fetal medicine fellows (31).

As compared to another previous study done in Ethiopia on similar setting the rate of adverse perinatal outcomes was found to be higher, LBW [19.32 vs. 11.5%], NICU admission [26.14 vs. 10.5%] &MSAF [23.11 vs. 12.88] (5). This can be explained by higher number of isolated oligohydramnios and elective cesarean delivery in the previous study. Alternatively, it may be explained by the previous study was conducted on term pregnancy as compared to ours which includes all 3rd trimester cases.

As compared to similar studies done in India and Texas (United States) the rate of adverse outcomes was found to be lower, LBW [19.32% vs. 26.6%–38.6%], NICU admission [26.14% vs. 24%–40%], NRFHR [25.38% vs. 48%] (3, 17, 32–33).

Parity, presence of hypertensive disorders of pregnancy, presence of intrauterine growth restriction was found to be associated with development of adverse perinatal outcome. This was similar with a study done in Ethiopia and India which reported hypertensive disorders and IUGR as associated factors (6, 34).

The high rate of adverse fetal outcome in nulliparous may be explained by increased risk for other obstetric problems like HDP (53.33% vs. 46.67%) and IUGR (57.63% vs. 42.67%) as compared to multiparous and labor abnormalities with its associated increased fetal risk. This may also be explained compared to previous studies done in Ethiopia in which the presence of these factors leading to elective and emergency cesarean delivery in our study they were allowed to labor unless they have emergency fetal indication.

## Strength and limitation of the study

Done on primary data where there is limited evidence on oligohydraminos associated adverse outcomes. However, the fact it is a cross-sectional study, it may not be sufficient enough to identify adverse outcomes and associated factors.

## Conclusion

This study found that mothers with oligohydramnios at 3rd trimester are at increased risk of developing adverse perinatal outcome. The presence of HDP, IUGR and nulliparous were found to be predictors of adverse perinatal outcome. However, development of adverse perinatal outcomes was not associated with type of labor and delivery (spontaneous, induced or elective CD).

## Data Availability

The original contributions presented in the study are included in the article/Supplementary Material, further inquiries can be directed to the corresponding author.
